# Chitosan-Poly (I:C)-PADRE Based Nanoparticles as Delivery Vehicles for Synthetic Peptide Vaccines

**DOI:** 10.3390/vaccines3030730

**Published:** 2015-09-11

**Authors:** Jorge F. Correia-Pinto, Noemi Csaba, John T. Schiller, Maria J. Alonso

**Affiliations:** 1 Department of Pharmacy and Pharmaceutical Technology, School of Pharmacy, University of Santiago de Compostela, 15782 Santiago de Compostela, Spain; E-Mails: jo.pinto84@gmail.com (J.F.C.-P.); noemi.csaba@usc.es (N.C.); 2 Center for Research in Molecular Medicine and Chronic Diseases (CIMUS), Av. Barcelona s/n, Campus Vida, University of Santiago de Compostela, 15707 Santiago de Compostela, Spain; 3 Laboratory of Cellular Oncology, National Cancer Institute, NIH, Bethesda, MD, USA; E-Mail: schillej@dc37a.nci.nih.gov

**Keywords:** peptide-based antigens, nanoparticle, adjuvant, chitosan, poly (I:C), HPV, T-Helper peptide

## Abstract

The safety and precision of peptide antigens has prompted the search for adjuvants capable of increasing the immune response against these intrinsically poorly immunogenic antigens. The integration of both immunostimulants and peptide antigens within nanometric delivery systems for their co-delivery to immune cells is a promising vaccination strategy. With this in mind, the potential synergistic effect of the immunostimulant poly (I:C) (pIC) and a T-Helper peptide (PADRE), integrated into a chitosan (CS) based nanostructure, was explored. The value of this nanostructured combination of materials was assessed for a peptide antigen (1338aa) derived from the HPV-16 L2 protein. These nanoparticles, produced by ionic gelation technique, exhibited a nanometric size (<300 nm), a high positive surface charge (>40 mV) and high pIC association efficiency (>96%). They also showed capacity for the association of both the 1338aa and PADRE peptides. The influence of the presence of pIC and PADRE in the nanocomposition, as well as that of the peptide presentation form (encapsulated *versus* surface adsorbed) on the antibody induction was evaluated in a preliminary *in vivo* study. The data obtained highlights the possibility to engineer nanoparticles through the rational combination of a number of adjuvant molecules together with the antigen.

## 1. Introduction

The advent of synthetic peptide-based antigens has brought new opportunities and also new challenges to the development of effective vaccines. These synthetic and chemically well-defined molecules, designed from known epitopes of tumor cells and pathogens, present no risk of mutation or reversion associated to whole pathogen-based vaccines. From the technological point of view, they are easy to produce under appropriate quality control practices [1] and present little risk of contamination by pathogenic or toxic substances [2]. However, due to their purity, these entities lack “danger signals” [3] and consequently are incapable of activating immune cells. Furthermore, peptide-based antigens present, in general, limited major histocompatibility complex (MHC) recognition, which contributes to their poor intrinsic immunogenicity [4]. Therefore, there is a need to design adjuvant systems specifically adapted to facilitate the efficient presentation of peptide-based antigens without compromising their favorable safety profile. Within this context, nanoparticles made of biodegradable polymers are receiving increasing attention.

Among the different nanotechnology-based adjuvant systems, nanoparticles made of chitosan (CS), a biodegradable polysaccharide [5], are particularly attractive since these nanoparticles have already proven to be efficient adjuvant systems for a variety of protein antigens [6,7]. In addition to their capacity for associating with peptides and proteins, CS nanoparticles are well known for their ability to efficiently complex polynucleotides, protect them from enzyme degradation, and deliver them to cells [8]. Therefore, in principle, such delivery systems could offer the possibility of associating simultaneously antigens and polynucleotide-based immunostimulants and delivering them to the target site in a controlled manner.

As a model peptide-based antigen that would potentially benefit from the chitosan-based nanotechnology, we have chosen the peptide (1338aa), which comprises a specific amino sequence (13–38aa) from the Human Papillomavirus 16 (HPV-16) L2 capsid protein. Currently available HPV vaccines are based on the L1 protein from the HPV capsid that self assembles into virus-like particles (VLPs) [9]. These vaccines induce high levels of type-specific neutralizing antibodies, however, these antibodies have a limited ability to protect against other oncogenic HPV types [10,11]. On the other hand, the L2 minor capsid protein does not assemble into VLPs and is less immunogenic but it contains conserved domains that elicit broad cross-type neutralizing antibodies [12], which makes L2 a potential target for a novel HPV vaccine. The amino acid sequence 17–36aa of this protein has been identified as a highly conserved neutralizing epitope among the HPV strains [13] and so has great potential as an antigen for a broadly cross-protective HPV vaccine.

In order to enhance the adjuvant properties of chitosan nanoparticles, the immunostimulant Polyinosinic-polycytidylic acid—poly (I:C) (pIC), a synthetic analog of viral double-stranded RNA (dsRNA), was incorporated into the nanostructure. This compound mimics a molecular pattern associated with viral infections and it is an agonist of the Toll-like receptor 3 (TLR3), present in the endosomes of antigen-presenting cells (APC). The activation of TLR3 promotes dendritic cell maturation and the production of T cell chemokines [14], thereby potentiating both humoral and cellular immune responses [15,16]. Nevertheless, the systemic delivery of pIC can also provoke adverse effects, such as autoimmunity [17] and chronic inflammatory diseases [18], as observed in rodent models. In the context of this work, it was hypothesized that the incorporation of this immunostimulant into CS nanoparticles could protect it from degradation and facilitate its uptake by the APCs, consequently leading to a reduction of its effective dose [19,20].

Finally, in addition to the HPV L2 antigenic peptide and the pIC immunostimulant, a T-Helper Pan HLA-DR epitope (PADRE) peptide was associated with the composition. This peptide can bind to the majority of MHC class II alleles [21] and has previously been shown to increase the immune response to associated peptide-based antigens [22,23]. Accordingly, the inclusion of the PADRE peptide in the nanoparticle is expected to provide linked T cell helper functions to the 1338aa peptide and, consequently, an increased immune response against the antigen.

To summarize, the objective of this work was to design and develop a CS based nanoparticle with the capacity to co-deliver a peptide-based antigen with the immunostimulants pIC (as danger signal) and the PADRE peptide (to increase T helper responses). The ultimate goal has been the identification of potential synergistic effects among the three major components with regard to their ability to generate an effective antibody response. The expected low immunogenicity of the L2 peptide 1338aa allied with its great potential as antigen for an improved HPV vaccine makes it an excellent antigen model for the study of novel particle-based vaccines with intrinsic adjuvant activities. Even though the synergistic effect of the combination of the T helper peptides and pIC for immunization purposes has already been observed for a peptide-based antigen from HPV [23], this is, to the best of our knowledge, the first time that both molecules are combined in nanoparticles as an adjuvant system to solve the specific limitations of peptide-based antigens.

## 2. Experimental Section

### 2.1. Materials

Peptide 1338aa and peptide PADRE were chemically synthesized (95% and 99% purity, respectively) by Genemed Synthesis (San Antonio, TX, USA). Amino acid sequence and physicochemical characteristics are described in Table 1. Polyinosinic-polycytidylic acid of low molecular weight (0.2–1 kB) (Poly (I:C)-LMW) was purchased from InvivoGen (San Diego, CA, USA). Ultrapure chitosan hydrochloride salt (Protasan UP Cl 113, Mw: 50,000–150,000 g/mol) was purchased from Novamatrix (Norway). Degree of deacetylation was confirmed by elemental analysis to be 75% ± 2%. Pentasodium tripolyphosphate (TPP), trehalose, TFA (Trifluoroacetic acid ≥99%), ammonium hydroxide and heparin were obtained from Sigma-Aldrich (Madrid, Spain). Chitosanase 10 U purified from *Streptomyces griseus* (C4163-01) was purchased from United States Biological (Salem MA, USA). SYBR Gold^®^ and Nunc Maxisorp™ MicroWell™ flat-bottom 96-well plates were purchased from Thermo Fisher Scientific (Waltham, MA, USA). HPLC-grade acetonitrile and water were purchased from Scharlab (Barcelona, Spain) and Fisher Chemical (Thermo Fisher Scientific, Waltham, MA, USA).

Human serum adsorbed and peroxidase labeled anti-mouse IgG (H + L) goat antibody was purchased from KPL (Gaithersburg, MD, USA). The secondary anti-Rabbit IgG horseradish peroxidase linked whole antibody from donkey was purchased from Amersham (GE Healthcare Life Sciences, Little Chalfont, UK). ABTS was acquired from Roche (Basel, Switzerland). Imject^®^ Alum (aluminum hydroxide) was obtained from Pierce Biotechnology (Waltham, MA, USA).

**Table 1:** Physicochemical characteristics of the peptides.

Peptide	Amino Acid Sequence	MW	Purity	pI	Charge at pH			
					5.7	6.4	8.8	9.6
1338aa	ASAWQLYKTCKQAGTCPPDIIPKVEG DDDDDD	3411	95%	3.94	−4	−4	−6	−7
PADRE	(d-Ala)K(Cha)VAAWTLKA(d-Ala) DDDDDD	1973	99%	3.85	−4	−4	−4	−5

MW: Molecular weight as provided by manufacturer; (Cha): Cyclohexylalanine; (d-Ala): d-Alanine; pI: Theoretical pI predicted by online software from SIB Swiss Institute of Bioinformatics [24]; Charge at pH: Charge calculated according to the method described in Appendix Equetion A1.

### 2.2. Design and Preparation of CS and pIC Based Nanoparticles

The nanoparticles were produced by the ionic gelation technique originally developed in our group [25] and extensively used for the association of peptides, proteins and pDNA [6,7,26,27,28,29]. According to this technique, the nanoparticles are formed spontaneously due to the ionic cross-linking of CS molecules by the TPP. In the current study, for the formation of the nanoparticles, pIC was also included in the TPP aqueous phase. In order to obtain a monodisperse population of nanometric particles with efficient pIC incorporation, different TPP and pIC concentrations were tested. The TPP concentrations were 0, 0.25, and 0.625 mg/mL while in the case of the pIC were 0.25, 0.625 and 1.25 mg/mL, with a constant volume of 0.2 mL. The CS concentration and volume were fixed at 1 mg/mL and 0.5 mL, respectively. The resulting nanoparticles were allowed to form for 30 min and then, they were isolated by ultracentrifugation at 20,000 RCF, 4 °C for 2 h (Centrifuge 5430R, Eppendorf AG, Hamburg, Germany) on a glycerol bed. The supernatant was removed and the pellet was resuspended in 0.1 mL of ultrapure water.

### 2.3. Physicochemical Characterization

Particle size, polydispersity index (PdI) and derived count rate (DCR) were evaluated by Dynamic Light Scattering (DLS), and zeta-potential by Electrophoretic Light Scattering (ELS) using a Zetasizer Nano ZS90 (Malvern Instruments, Worcestershire, UK). The measurements were performed at 25 °C with a detection angle of 173°, in distilled water. The morphology of the nanoparticles was examined by transmission electron microscopy (TEM) (CM 12 Philips, Eindhoven, The Netherlands). The nanoparticles were placed on copper grids with Formvar films and stained with 2% (w/v) phosphotungstic acid solution. The grids were left overnight in an oven at 60 °C to dry and then observed with TEM. The solutions and formulation pH was determined with a Sartorius Docu-pH Benchtop Meters (Thermo Fisher Scientific).

### 2.4. Evaluation of pIC Loading in Nanoparticles

The association efficiency of pIC into the nanoparticles was calculated indirectly, from the amount of free pIC detected in the supernatant collected upon ultracentrifugation. The free pIC was determined by absorbance at 260 nm (Abs 260 nm) with a NanoDrop 2000 (Thermo Fisher Scientific) and quantified by interpolation in a linear standard curve (*R*^2^ = 0.9988) produced with pIC solubilized in the supernatant of blank nanoparticles in a concentration range from 2.5 to 80 μg/mL.

The association of pIC was confirmed by electrophoresis in a 1% agarose gel using a Sub-Cell GT 96/192 electrophoresis system (Bio-Rad, Hercules, CA, USA). Briefly, the isolated nanoparticles were stained with SYBR Gold^®^ and then 0.025 mL of formulation was added (equivalent to 8.4 μg pIC) to the agarose gel wells. After applying 90 V for 45 min, the gels were evaluated with an UV transilluminator (Molecular Imager^®^ Gel Doc™ XR, Bio-Rad, CA, USA) and analyzed with Image Lab™ Software (Bio-Rad, Hercules, CA, USA).

The reversibility of the pIC association was tested by incubating the nanoparticles with heparin (Sigma-Aldrich, Madrid, Spain) and verifying its release in an agarose gel [26]. In brief, fresh isolated nanoparticles and freeze dried nanoparticles were incubated in a 1.2 mg/mL heparin solution for 2 h at 37 °C in order to have an excess of heparin in relation to the pIC (mass ratio pIC/heparin 1/3) and induce the competitive displacement of the pIC molecules by the anionic polysaccharide.

In order to verify that pIC remained intact upon incorporation into the CS matrix, the nanoparticles were incubated with chitosanase (an enzyme capable of hydrolyzing the CS into oligosaccharides, releasing the associated pIC) [7,26]. For this, 0.1 mL of fresh isolated nanoparticles (0.4608 mg) were incubated with 1.1 U of chitosanase in a 50 mM acetate buffer pH 5.5, for 4 h at 37 °C under agitation. After incubation, an aliquot (0.019 mL) was taken and tested in a 1% agarose gel. This same protocol was also applied to freeze dried particles (described below) to verify pIC stability in the nanoparticles after the freeze drying process.

### 2.5. Peptide Association to Nanoparticles

**Figure 1:**
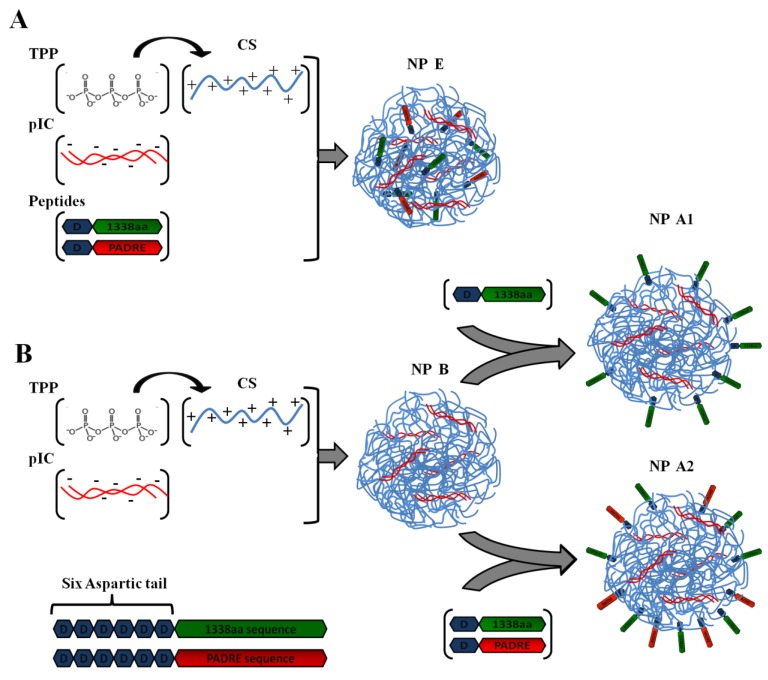
Illustration of peptide-loading into nanoparticles either by encapsulation of both peptides to generate the formulation NP E (**A**); by adsorption of 1338aa or of 1338aa and PADRE to generate the formulation NP A1 and NP A2, respectively (**B**). Representation of both peptides, divided in their active part (1338aa sequence and PADRE sequence) and its six aspartic acid segment designed to promote their interaction with the CS amino groups.

Firstly, the 1338aa peptide was solubilized in sterile water at the concentration of 10 mg/mL, while the PADRE peptide, due to its higher hydrophobicity, was solubilized in a 0.07 M ammonium hydroxide solution to the final concentration of 5 mg/mL. The association of the peptides to the CS/TPP/pIC nanoparticles was achieved either by incorporation of the peptides into the TPP/pIC containing phase (Protocol A), during the nanoparticles’ formation process (Section 2.2.), or by their incubation with the preformed nanoparticles (Protocol B), as illustrated in Figure 1.

In Protocol A, all components to be entrapped within the CS matrix, poly (I:C), PADRE peptide and 1338aa peptide were incorporated into the aqueous phase containing TPP. The concentration of these ingredients in the aqueous phase was: 0.625 mg/mL for TPP, 0.25 mg/mL for poly (I:C), 0.125 mg/mL for PADRE peptide and 0.125 mg/mL for 1338aa peptide. The final pH of this phase upon dissolution of all these ingredients was 8.8. Then, a volume of 0.2 mL of this aqueous phase was added to a 0.5 mL volume of the CS phase (1 mg/mL, pH 4.80) and the mixture was maintained under magnetic stirring for 5 min to produce the formulation NP E.

In Protocol B, the peptide was adsorbed onto the preformed nanoparticles. These nanoparticles were produced by mixing 0.2 mL of the aqueous phase containing TPP and pIC (0.625 and 0.25 mg/mL, respectively) with 0.5 mL of the CS phase (1 mg/mL). After the nanoparticles were formed, a 0.1 mL of 1338aa peptide aqueous solution (0.25 mg/mL) containing the PADRE peptide (0.25 mg/mL; pH 9.6) or not (pH 6.4) was poured over 0.7 mL of the nanoparticle suspension (pH 5). The system was maintained under magnetic stirring for 5 min and, then, the formulation was left to stand for 1 h at room temperature. The resulting nanoparticles were named as NP A2 and NP A1, depending on whether they included PADRE in their composition or not. After the incubation period, the nanoparticles were isolated by ultracentrifugation at 20,000 RCF, 4 °C for 2 h on a glycerol bed (Centrifuge 5430R, Eppendorf AG, Germany). An additional nanoparticle composition was produced according to the protocol used NP A2, but without pIC (NP C). The theoretical loading of each peptide with respect to the CS mass was 5% in all cases.

### 2.6. Evaluation of the 1338aa and PADRE Association

To evaluate the peptide association efficiency, the loaded nanoparticles were first isolated by ultracentrifugation, as previously described. The supernatant was eliminated and the nanoparticles pellet was recovered and digested with chitosanase in the same conditions as reported above (Section 2.4). The peptide released after nanoparticle digestion was determined by an ultra-performance LC (UPLC) coupled with UV detection system (ACQUITY UPLC H-Class, Milford, MD, USA) using a reverse phase Acquity UPLC BEH C18 column (1.7 um 130 Å 2.1 × 100 mm) and a gradient elution. The mobile phase A consisted of TFA 0.1% (v/v) aqueous solution and the mobile phase B of TFA 0.1% (v/v) in acetonitrile. Other UPLC conditions are described in Appendix Table A1. For the standard curve, the peptides were diluted in the digested matrix of nanoparticles (NP B) at concentrations between 3.125 and 100 μg/mL.

### 2.7. Freeze-Drying of Nanoparticles

Lyophilization was conducted in a VirTis Genesis 25L lyophilizer (Model SQ EL-85, SP Scientific, Warminster, PA, USA). The samples were frozen overnight at −20 °C and then transferred to the lyophilizer. They underwent an initial drying step for 24 h at −35 °C at 2–10 millitorr (mtorr) followed by a secondary drying for another 24 h at 0 °C and finally a third step of 16 h at 20 °C at the same pressure. Trehalose was used as a cryoprotectant at different concentrations (0%, 2.5%, 5% and 10% w/v), while the nanoparticle concentration was evaluated at 42% and 25% (v/v). After freeze drying, loaded nanoparticles were reconstituted with ultrapure water (0.1 mL) in order to analyze their particle size and PdI. The same freeze-drying cycle was used to dehydrate the isolated formulations and determine the formulation process yield and the nanoparticle concentration by gravimetric analysis.

### 2.8. Immunization Studies

The ability of nanoparticles to induce an antibody response against the 1338aa peptide was assessed in 6- to 8-week-old female BALB/c mice obtained from the National Cancer Institute (NCI), housed and handled under specific pathogen-free conditions in the animal care facilities at the NCI, Bethesda. Groups of 5 mice were randomly assigned and immunized intramuscularly (i.m.) in the anterior thigh, with a first and second boost given on day 21 and 42, respectively. The immunization dose of 1338aa was 5 μg (0.05 mL).

The control, alum-adsorbed antigen, was prepared according to the manufacturer’s specifications. Briefly, the Imject^®^ Alum was agitated until complete homogenization of the dispersion and then it was added dropwise over a 1338aa solution in PBS, producing a final dispersion of alum salt and 1338aa at the concentration of 0.1 mg/mL. This dispersion was left under agitation for 1 h at room temperature before administration. As an additional control, the same formulation without pIC (NP C) was used.

The blood samples (0.2 mL) were collected by retro-orbital bleeding at 0, 3, 6 and 8 weeks after the first vaccination. The animals were anesthetized (isofluorane 5% in oxygen vapor) before each immunization and blood sample collection. The blood samples were ultracentrifugated for 10 min at 14,926 RCF and then 0.05 mL of serum was collected. Serum IgG levels for 1338aa were determined for each sample by ELISA. For this purpose, Maxisorp^®^ 96-wells were coated with 0.1 mL of the 1338aa peptide at 5 μg/mL in PBS and incubated overnight at 4 °C. After washing with PBS, the wells were blocked with 0.2 mL of 5% bovine serum albumin in carbonate-bicarbonate buffer for 2 h at 37 °C. After washing again with PBS, 0.1 mL of the respective serum, diluted 1:50 in PBS with 2% fetal bovine serum (FBS), was added and incubated 1 h at 37 °C. After new wash with PBS and 0.05% Tween 20, 0.1 mL of secondary antibody (goat anti-Mouse IgG (H + L) labeled with HPR) diluted 1:1000 in PBS with 1% FBS was added to the wells, and left incubating for 1 h at room temperature. Finally, the wells were washed again with PBS, and then the ABST substrate was added to the wells. After 40 min incubation, the absorbance was determined in a microplate reader (405 nm, POLARstar OPTIMA, BMG-Labtech Gmbh, Ortenberg, Germany). All the washing and blocking steps were done with an automated microplate washer (ELx405 Select Deep Well Microplate Washer, Biotek, Winooski, VT, USA). All serum samples were tested in duplicate. As positive control for the 1338aa peptide coating of the wells, HPV16 L2 peptide 17–36aa rabbit antiserum was used in a dilution of 1:1000 [13]. As secondary antibody, an anti-rabbit IgG was used in a dilution of 1:2000.

## 3. Results and Discussion

The objective of the present work was to design a nanocarrier, specifically adapted for the co-association of antigens and different types of immunostimulants. The final goal was to explore the synergistic effect of different adjuvant molecules assembled together in a nanostructure. With this idea in mind, three main components were chosen for the formation of the nanostructures: chitosan, known for its capacity to facilitate antigen presentation to the immune system when presented in a nanometric structure [6,7], poly (I:C), a potent immunostimulant, and a T-Helper peptide (PADRE). In addition, these nanoparticles were expected to protect poly (I:C) from degradation [30], and facilitate its delivery in the immune cells, where its receptor (TLR3) is located. To assess the value of this nanocomposition, a challenging HPV related peptide-based antigen (1338aa) was selected (Figure 1). This peptide was either adsorbed onto the nanoparticles surface, or incorporated within the nanoparticle structure. The key aspects of the development and characterization of these new nanostructures and the results of an exploratory *in vivo* efficacy assay are reported below.

### 3.1. Design and Characterization of Chitosan-Poly (I:C) Based Nanoparticles

The development process of the multicomponent nanoparticles consisted of several subsequent steps. The first step involved the entrapment of poly (I:C) into CS nanoparticles. Based on previous work from our group [25], a CS/TPP mass ratio of 4/1 and 8/1 was selected for the formation of the nanoparticles. The TPP is an ionic cross-linking agent that promotes the gelation of chitosan and facilitates the formation of well-defined spherical nanoparticles. The CS/pIC mass ratio tested was 4/2, 4/1 and 4/0.4, which is equivalent to a pIC theoretical loading of 10%, 25% and 50%, respectively, in relation to the total amount of CS used for particle preparation (Table 2). Simple complexes of CS and pIC, without TPP, were also produced as previously described [26]. The positive-to-negative charge (P/N) ratio, defined as the ratio between the maximum number of protonable primary amines in CS (based on a 75% deacetylation degree, as determined by elemental analysis) and the sum of negative phosphate groups from TPP and pIC is also presented in Table 2 for each composition.

As shown in Table 2, the CS/TPP/pIC mass ratio 4/1/1 and 4/1/2 (25% and 50% pIC loading, respectively) led to the formation of aggregates or highly polydisperse particles. This could be related to the fact that the P/N ratio of these formulations was close to neutrality (1/1.13 and 1/0.95) and/or to the formation of additional complexation species, e.g., complexes of CS and p(I:C). In fact, in agreement with previous findings [25], the complexes formed by ionic interaction of CS and pIC without TPP presented a high PdI as compared with nanoparticles produced by ionic gelation of CS with TPP. The entanglement of the pIC in the gelled matrix of CS and TPP may provide a better entrapment of the polynucleotides in comparison to the simple electrostatic interaction between CS and pIC, as observed previously for pDNA [26] and dsRNA [30]. Thus, the gelled nanostructure could be more adequate for preventing the premature release of pIC. This is a critical issue given the potential toxicity associated to the systemic release of pIC [20].

**Table 2:** Physicochemical characterization of Chitosan-Poly (I:C) nanoparticles at different mass ratios.

CS/TPP/pIC	P/N	pIC (%)	Size (nm)	PdI	ζ (mV)	Derived Count Rate
4/1/2	1/1.13	50	Aggr.	-	-	-
4/1/1	1/0.95	25	369 ± 27	0.5	+32 ± 3	108264
4/1/0.4	1/0.85	10	284 ± 15	0.2	+41 ± 1	46537
8/1/4	1/0.74	50	363 ± 14	0.3	+42 ± 2	64724
8/1/2	1/0.56	25	290 ± 25	0.2	+43 ± 5	24955
8/1/0.8	1/0.46	10	302 ± 23	0.2	+55 ± 3	10495
8/0/4	1/0.35	50	350 ± 24	0.3	+49 ± 1	49503
8/0/2	1/0.17	25	243 ± 8	0.3	+56 ± 1	19691
8/0/0.8	1/0.07	10	172 ± 5	0.2	+56 ± 1	6064

CS/TPP/pIC mass ratio: Mass ratio between chitosan, TPP and poly (I:C); P/N charge ratio: Charge ratio between the amine groups of CS and the sum of the phosphate groups of TPP and pIC; pIC Loading (%): Loading of poly (I:C) in particles in relation to the chitosan mass; PdI: Polydispersity Index; ζ: Zeta-potential. Results are presented as mean ± standard deviation (*n* = 4).

**Figure 2:**
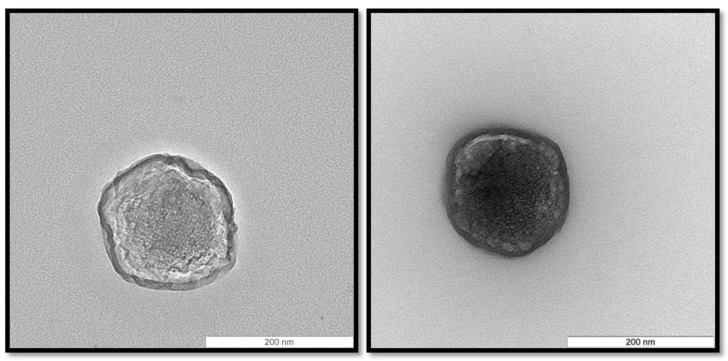
Morphology of Chitosan/TPP/Poly (I:C) nanoparticles (NP B) visualized by transmission electron microscopy. The scale bars correspond to 200 nm.

Among the CS/TPP/pIC mass ratios tested, those consisting of 4/1/0.4 and 8/1/2 led to the formation of nanoparticles with an acceptable size (<300 nm) and PdI (<0.3). On the other hand, the yield of the nanoparticles formation process was much higher for the ratio 4/1/0.4 than for 8/1/2 (yield of 77 and 47%, respectively). Therefore, the CS/TPP/pIC mass ratio 4/1/0.4 (from now on named NP B) was selected for further experiments. These nanoparticles exhibited a spherical shape (Figure 2) and a high positive surface charge (+41 mV). This positive charge, which indicates that pIC was conveniently entrapped within the chitosan matrix, is an important feature for the subsequent adsorption of the anionic peptides 1338aa and PADRE and also to facilitate nanoparticle uptake by antigen-presenting cells [31].

### 3.2. Association and Release of pIC from CS/TPP/pIC Nanoparticles

**Figure 3:**
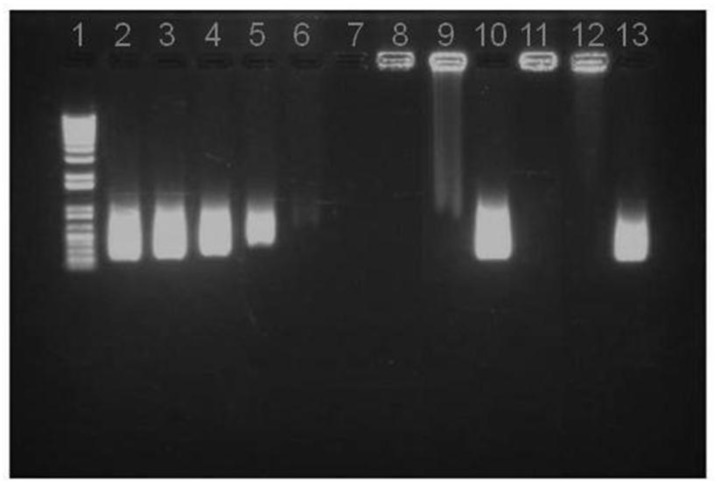
Agarose gel assay on pIC release from NP B (Chitosan/TPP/Poly (I:C) without peptides): (**1**) DNA Ladder; (**2**) 8 μg pIC; (**3**) 4 μg pIC; (**4**) 2 μg pIC; (**5**) 1 μg pIC; (**6**) 0.5 μg pIC in solution; (**7**) NP B Supernatant; (**8**) NP B Pellet; (**9**) NP B Pellet incubated with heparin; (**10**) NP B Pellet digested with chitosanase; (**11**) NP B Pellet freeze dried; (**12**) NP B Pellet freeze dried and incubated with heparin; (**13**) NP B Pellet freeze dried incubated with chitosanase.

As determined by spectrophotometry, the association efficiency of pIC in the selected mass ratio composition CS/TPP/pIC 4/1/0.4 was close to 100%. This high pIC association efficiency was further confirmed by agarose gel electrophoresis. The migration patterns presented in Figure 3 show the absence of bands corresponding to free pIC in the nanoparticles suspending medium (obtained upon centrifugation of the suspension) (well 7) or in the nanoparticle suspension (well 8). This high efficiency is in agreement with previous work showing the high capacity of CS/TPP nanoparticles for the association of DNA and dsRNA [26,30]. To verify if the pIC was firmly associated to the nanoparticles, the system was incubated with heparin, a strong polyanion that might compete for the CS amino groups and displace loosely attached polynucleotide material [32]. The results showed that after incubation with an excess of heparin (1.2 mg/mL), only a small amount of pIC migrated (well 9), which illustrates the high affinity of pIC towards CS/TPP nanoparticles. This is an important result as it is known that the cell extracellular matrix contains polyanions similar to heparin that might favor the dissociation of polynucleotides [33]. In order to assess the stability of the complexed pIC, the nanoparticles were digested with chitosanase to facilitate the release of pIC, which was then evaluated by agarose gel electrophoresis. The results showed that the migration pattern of the released pIC molecules was the same as that of the control pIC. Consequently, it can be concluded that pIC was firmly associated to the nanoparticles and could be released in an appropriate manner as a result of the polymer degradation. These results are in agreement with those previously reported showing the capacity of chitosan/TPP nanoparticles to deliver DNA [26] and dsRNA to different cells [30], including macrophages [34]. However, from our knowledge, this is the first report disclosing the potential of chitosan nanoparticles for the association of pIC as a strategy for enhancing the immunogenic response to the associated antigens.

### 3.3. Evaluation of the 1338aa and PADRE Peptide Association to CS/TPP/pIC Nanoparticles

The peptides 1338aa and PADRE were designed to encode HPV16 L2 neutralizing and T-helper amino acid sequences, respectively. In addition to these active sequences, a segment of six aspartic acid molecules was also incorporated at the C-terminus region of both peptides (Table 1). This segment was intended to work as an electrostatic anchor for the positively charged chitosan molecules, thus leaving the active region of the peptide available for interaction with its specific receptors (Figure 1). Based on the calculation described in Appendix Equation A1, at the formulation pH (5.74), the peptides are expected to present a high negative charge, as shown in Table 1, which would enable the electrostatic interaction with the positively charged nanoparticles (Table 2).

Two different protocols were used for the association of the peptides to the nanoparticles in order to promote their localization, either inside (Protocol A) or on the surface of the nanoparticles (Protocol B) (Figure 1). The nanoparticles obtained by both protocols, presented a nanometric size and low PdI (Table 3). In both methods, the resulting peptide-loaded nanoparticles presented a positive surface charge, which in the case of the particles produced by the Protocol B indicates that their surface was not totally covered by the adsorbed peptide molecules. In Protocol A, the peptides were dissolved together with TPP and pIC and added to the CS phase to facilitate simultaneous 1338aa and PADRE entrapment within the nanoparticle matrix (loading efficiency of 67% and 71%, respectively) (Table 3). The basic pH of the TPP/pIC/peptides phase (pH 8.8) possibly increased the anionic character of the peptides (Table 1), thereby improving their interaction with CS. In Protocol B, the peptides were simply adsorbed onto the nanoparticle surface and, as expected, the L2 antigen association efficiency was lower (39%–50%) [26], although the PADRE association remained almost unaltered (75%). Interestingly, when the peptide 1338aa was adsorbed onto the nanoparticles in combination with the PADRE peptide, it had higher association than added alone (50% and 39%, respectively) (Table 3). A possible cause for this could be that the combined solution of 1338aa and PADRE had a higher pH (9.6) than that of the 1338aa peptide alone (6.4). This higher pH results from the fact the PADRE was solubilized in a 0.07 M ammonium hydroxide. This could have increased the negative charge of 1338aa (Table 1) and, consequently, promote interaction with the positively charged CS.

**Figure 4:**
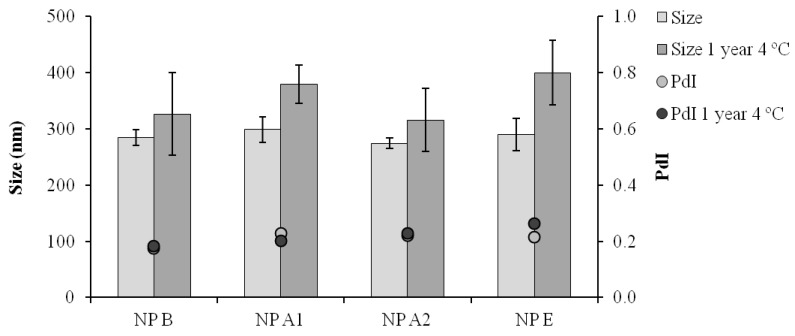
Particle size distribution (Size) and polydispersity index (PdI) after one year storage at 4 °C in suspension; NP B: Chitosan/TPP/Poly (I:C) nanoparticles without peptides; NP A1: Chitosan/TPP/Poly (I:C) nanoparticles with 1338a adsorbed at surface; NP A2: Chitosan/TPP/Poly (I:C) nanoparticles with 1338aa and PADRE adsorbed at surface; NP E: Chitosan/TPP/Poly (I:C) nanoparticles with 1338aa and PADRE encapsulated. Results are presented as mean ± standard deviation (*n* = 3).

**Figure A1:**
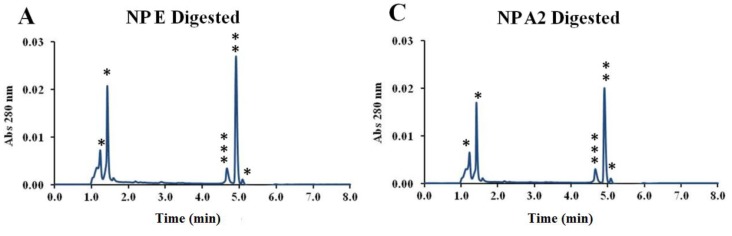

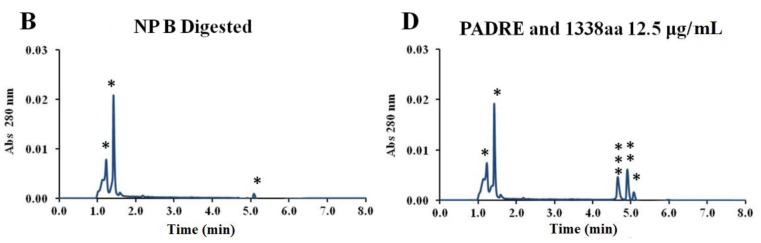
Comparison between UPLC peaks from digested blank nanoparticles and peptide-loaded nanoparticles, and the free PADRE and 1338aa peptides in digested nanoparticle matrix. Comparison between UPLC peaks of digested peptide-loaded nanoparticles (**A**) encapsulated (**B**) surface adsorbed) with blank nanoparticles (**C**) and with free PADRE and 1338aa peptides in digested nanoparticle matrix as controls (**D**); *: Peaks from residues of digested nanoparticles; **: Peak from PADRE peptide; ***: Peak from 1338aa peptide.

By digesting the nanoparticles and analyzing the released peptides by UPLC, it was also possible to verify that both peptides maintained their stability upon association to the nanoparticles (Appendix Figure A1). The mild formulation conditions probably contributed to the stability observed both for the pIC and peptides associated to the nanoparticles. In addition, all the formulations maintained their physicochemical characteristics for at least one year at 4 °C (Figure 4).

**Table 3:** Physicochemical characteristics and loading efficiency of Chitosan/TPP/Poly (I:C) nanoparticles (Chitosan/pIC) loaded with 1338a and PADRE peptides.

Formulation	Code	Size (nm)	PdI	ζ (mV)	1338aa (%)	PADRE (%)
Chitosan/pIC without peptide (Blank)	NP B	284 ± 15	0.2	+41 ± 1	N/A	N/A
Chitosan/pIC-1338aa Adsorbed	NP A1	299 ± 23	0.2	+49 ± 5	39 ± 1	N/A
Chitosan/pIC/PADRE-1338aa Adsorbed	NP A2	274 ± 9	0.2	+47 ± 5	50 ± 5	75 ± 13
Chitosan/pIC/PADRE-1338aa Encapsulated	NP E	290 ± 28	0.2	+48 ± 4	67 ± 8	71 ± 3

Chitosan/pIC-1338aa Adsorbed: Chitosan/TPP/Poly (I:C) nanoparticle with 1338aa adsorbed at surface; Chitosan/pIC/PADRE-1338aa Adsorbed: Chitosan/TPP/Poly (I:C) nanoparticle with PADRE and 1338aa adsorbed at surface; Chitosan/pIC/PADRE-1338aa Encapsulated: Chitosan/TPP/Poly (I:C) nanoparticle with PADRE and 1338aa encapsulated; Size: Particle size distribution; PdI: Polydispersity Index; ζ: Zeta-potential; 1338aa Loading (%): Percentage of peptide 1338aa associated to the nanoparticle; PADRE Loading (%): Percentage of peptide PADRE associated to the nanoparticle. Loading values were obtained from the direct determination of the peptide released after the digestion of the nanoparticles with chitosanase. Results are presented as mean ± standard deviation (*n* = 3).

### 3.4. Freeze-Drying of CS/TPP/pIC Nanoparticles

**Figure 5:**
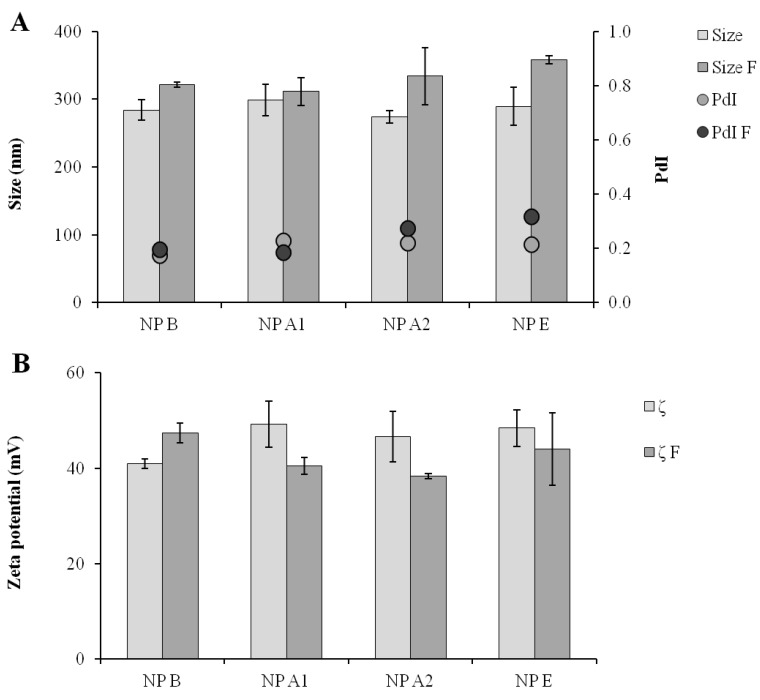
Comparison of the formulations in terms of particle size and polydispersity (**A**) and of zeta-potential (**B**) before and after freeze-drying and resuspension operation. Size: Size of the formulation before freeze-drying; Size F: Size after freeze-drying; PdI: Polydispersity of formulation before freeze-drying; PdI F: PdI after freeze-drying; ζ: Zeta-potential of formulation before freeze-drying; ζ F: Zeta-potential after freeze-drying; NP B: Chitosan/TPP/Poly (I:C) nanoparticles without peptides; NP A1: Chitosan/TPP/Poly (I:C) nanoparticles with 1338aa adsorbed at surface; NP A2: Chitosan/TPP/Poly (I:C) nanoparticles with 1338aa and PADRE adsorbed at surface; NP E: Chitosan/TPP/Poly (I:C) nanoparticles with 1338aa and PADRE encapsulated. Results are presented as mean ± standard deviation (*n* = 3).

In general, the dehydration of the nanoparticle formulations helps to prevent particle aggregation and degradation [35]. For this reason, the feasibility of freeze-drying the CS/TPP/pIC nanoparticles was explored. Trehalose was chosen as a cryoprotectant [35], and the influence of the formulation concentration (42% and 25% (v/v)) and the trehalose concentration (10%, 5% and 1% (w/v)) in the resuspension of the dried formulation was studied. The resulting powder cakes were resuspended with 0.1 mL of ultrapure water to provide for a final antigen concentration of 0.1 mg/mL. A 42% (v/v) nanoparticle concentration at 5% trehalose (w/v) generated an adequate cake, which could be easily resuspended. The resuspended particles presented only a modest variation in size, PdI and zeta-potential (between 10% and 15%), as shown in Figure 5. An additional positive effect of the developed freeze-drying method is that due to the final trehalose concentration (12%), the resuspended solution was close to isotonic.

The stability of the pIC associated to the freeze-dried nanoparticles upon one week of storage at room temperature was analyzed by agarose gel electrophoresis (Figure 3). Similar to the fresh particles, the absence of the free pIC band in the gel indicates that the pIC remains firmly attached to the particle after the freeze-drying (well 11) even after incubation with heparin at 37 °C (well 12). Furthermore, upon digestion of the nanoparticle with chitosanase, the pIC band became visible with a migration pattern and intensity (well 13) similar to those of the control (well 2). This indicates that these nanoparticles can efficiently protect the pIC from degradation during freeze-drying and upon storage in a dry powder form. After confirming the suitability of the freeze drying procedure for the pIC-containing nanoparticles, the peptide-loaded CS/TPP/pIC formulations were freeze-dried in the same conditions. The results showed that these formulations also preserved their physicochemical characteristics after resuspension, with only minor variation in size and zeta-potential (Figure 5).

### 3.5. Immunization Studies

**Figure 6:**
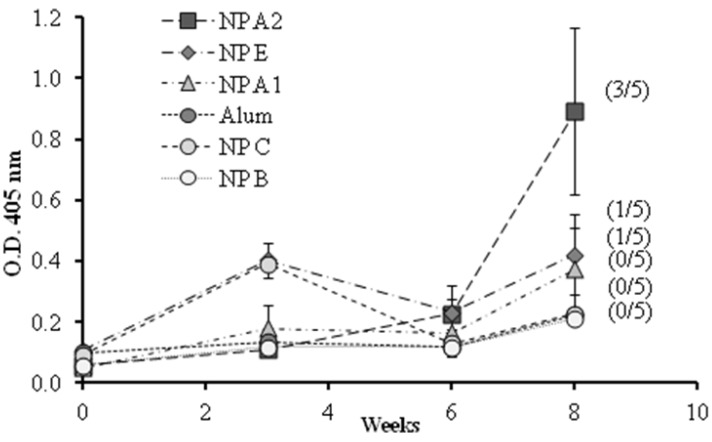
1338aa peptide-specific IgG responses in diluted serum (1/50) of mice immunized with different formulations by intramuscular route. NP B: Chitosan/TPP/Poly (I:C) nanoparticles without peptides; NP C: Chitosan/TPP nanoparticles with 1338aa and PADRE adsorbed on their surface; NP A1: Chitosan/TPP/Poly (I:C) nanoparticles with 1338aa adsorbed on their surface; NP A2: Chitosan/TPP/Poly (I:C) nanoparticles with 1338aa and PADRE adsorbed on their surface; NP E: Chitosan/TPP/Poly (I:C) nanoparticles with 1338aa and PADRE encapsulated. (Number/5): Number of responsive mice in the total five mice per group. An O.D. four times higher than those of the control NP B (0.2) was considered a positive response. Results are presented as mean ± standard error of mean (*n* =5).

A preliminary vaccination study was conducted to evaluate the influence of the different components and their organization on the ability of the nanoparticle to generate a humoral immune response. The formulations selected, presenting different 1338aa and PADRE arrangements were: nanoparticles with the peptide 1338aa alone adsorbed at the surface (NP A1), with the peptide 1338aa and PADRE both adsorbed at the surface (NP A2), and the nanoparticles with both peptides encapsulated (NP E). These formulations and also three controls, the alum-adsorbed antigen, nanoparticles without peptides (NP B) and nanoparticles with peptides but without pIC (NP C), were administered to mice by the intramuscular route and the IgG response was monitored for up to eight weeks (Figure 6).

The O.D. values for IgG shown in Figure 6 indicate that alum was unable to enhance the immune response against the 1338aa peptide. On the other hand, peptide-loaded CS nanoparticles without pIC, known for their capacity to increase IgG responses against various protein antigens [6,7,27,29,36,37], failed to generate peptide-specific IgG responses. These results underline the difficulty in generating an immune response against poorly immunogenic peptides. With regard to the behavior of the formulation adjuvanted either with pIC, PADRE or both, the results were variable, exhibiting from one to three responding animals out of five immunized mice. Among the formulations tested, the composition containing both pIC and PADRE was capable of generating significant IgG levels in three out of five mice. These results suggest that both T helper and immunostimulant signals are required in order to achieve satisfactory antibody responses. In addition, the display of the peptide on the nanoparticle surface also appears to be critical. Indeed, the number of responding mice was greater when both peptides, 1338aa and PADRE, were adsorbed rather than entrapped.

The HPV L2 epitope 17–36aa [13], present in the peptide 1338aa, has already been tested in mice [38] although at a higher antigen dose (40 μg). In this study, it was observed that the Freund`s adjuvant was unable to stimulate an effective immune response against this peptide, confirming the low immunogenicity of this antigen. However, the conjugation of this peptide to other T Helper peptide (P25) and to the immunostimulant Pam_2_Cys (a TLR2 agonist) was very effective at inducing peptide-specific IgG responses. The comparison of these previous data with those obtained in the current study led us to formulate different hypotheses. First, there is the possibility that either the antigen (17–36aa) or the immunostimulants (P25 and Pam2Cys), or their combination were more appropriate for enhancing the immune response. In this regard, it is important to keep in mind that, although sharing the same epitope, the peptide 1338aa presents a longer amino acid chain and an additional six aspartic segment in the C-terminus region, which could potentially lead to a different processing and presentation by the immune cells [3]. A second hypothesis is that the interactions of 1338aa with the nanoparticle preclude an effective interaction with the B cell receptors. A third possibility is that the conjugation of the T helper peptide to the antigen facilitates induction of T helper functions.

The poor B cell immunogenicity of peptide-based antigens has been associated, among other factors, with their inability to establish a highly repetitive epitope pattern, which results in the limited activation of naive B cells after engagement of their cell surface immunoglobulins [39]. It was hypothesized that the adsorption of the peptide 1338aa onto the surface of the nanoparticles would display the target antigen in a repetitive pattern on a nanometric structure, resembling a virus. This hypothesis was partially confirmed by the higher antibody response elicited by the prototypes with adsorbed antigen (NP A2), as compared to the prototypes entrapping the same (NP E). However, further improvements in the presentation of the peptide epitopes can be envisioned. As the target epitope of the peptide was connected directly with the six aspartic segments used for adsorption to the nanoparticle, it is possible that part of the active region became embedded in the nanoparticle matrix and hindered its recognition by the B cell receptors. The addition of a generic peptide spacer between the particle binding motif and the target peptide might overcome this problem and provide for a more efficient presentation of the target peptide to B cells. It could also be interesting to examine whether including PADRE and the target antigen in a single peptide would increase the T helper response and thereby increase antibody responses. Furthermore, while CS/TPP nanoparticles have demonstrated capacity for sustained release of proteins [7,27], the release profile with the peptides used herein has not been studied, and should be explored in detail in further studies. Finally, according to the data presented, and in agreement with previous studies [26,40], the pIC is firmly associated to the nanoparticles and so the timing of its release may not be optimal for immune activation. As indicated previously, the introduction of pIC in the nanoparticles was expected to potentiate DC maturation [14] under the assumption that it would be released in endocytic vesicles along with the bound peptides. It has already been observed that for an optimal immune response with pIC, it is important that its delivery occurs simultaneously with that of the antigen of interest [41]. On the other hand, it has been recently suggested that the association of immunostimulants with CS nanosystems might prevent their interaction with the target [42]. Such hypothesis should be clarified in further experiments in order to improve the adjuvant effect of the developed system.

Therefore, this study illustrates the technical feasibility of producing CS-based nanoparticles vaccines that incorporate peptide-based antigens in conjunction with immunostimulatory molecules, and provides insight into the complexity of the engineering process. A larger array of CS nanoparticles that incorporate different peptide and immunostimulant combinations and that vary in their structural organization and size will need to be developed in order to fully assess the value of this nanotechnology platform for generating effective peptide-based vaccines.

## 4. Conclusions

Here, we report for the first time, a chitosan nanostructure designed to co-deliver a TLR3 agonist (Poly (I:C)) and a T-helper peptide (PADRE) as adjuvant system for a HPV-derived peptide antigen (1338aa). These nanoparticles are produced by ionic gelation, a mild and gentle method, which permits by subtle modifications of the formulation procedure, either the encapsulation or the adsorption of the target peptides. The capacity of combining different materials (polysaccharides, polynucleotides and peptides) in the form of nanoparticles, and the possibility to freeze-dry them highlights the versatility of this system and opens the possibility of integrating other immune stimulants. The ability of these nanoparticles to induce an immune response towards a particularly poorly immunogenic antigen is in contrast to the low efficacy observed in the same study using alum salts adjuvant. In conclusion, the preliminary data presented here shows the potential of these nanoparticles as combined antigen/adjuvant structures to induce antibody responses to peptide antigens.

## Appendix

**Table A1:** UPLC conditions and peptide retention times.

Gradient			Additional Features		Retention Time (min)	
Time (min)	Phase A	Phase B	Flux (mL/min)	0.2	1338aa in dig.	4.674
0	80	20	Wavelength Abs (nm)	280	PADRE in dig.	4.927
1.50	80	20	Column Temp. (°C)	35 °C		
1.51	55	45	Filter time Constant	Normal		
3.00	80	20	Volume injected (uL)	5		

1338aa in dig.: 1338aa dissolved in Chitosan/TPP/Poly (I:C) nanoparticle digested matrix; PADRE in dig.: PADRE dissolved in Chitosan/TPP/Poly (I:C) nanoparticle digested matrix.

## Equation A1. Calculation of the peptides charge at specific pH.

Calculation was realized with the following equation:

Z = ∑_i_ Ni (10^pKai^/(10^pH^ + 10^pKai^)) – ∑_j_ Nj (10^pH^/(10^pH^ + 10^pKaj^))

in which Z represents the net charge of the peptide sequence, Ni the number of arginine, lysine and histidine residues and the N-terminus, and pKai, their pKa. The Nj represents the number of aspartic acid, glutamic acid, cysteine and tyrosine residues and *C*-terminus, and the pKaj, their pKa. Note: In both predictions, the non-standard residue ciclohexylalanine had to be ignored and the d-Alanine was replaced by the l-Alanine for calculation purposes, which increases the error. However, both amino acids are neutral so the influence in the pI and charge according pH should be negligible. On the other hand, the aspartic tail should always be quite dominant in terms of peptide charge properties.
